# Combination Therapy with Nintedanib and Sarilumab for the Management of Rheumatoid Arthritis Related Interstitial Lung Disease

**DOI:** 10.1155/2020/6390749

**Published:** 2020-03-09

**Authors:** Caterina Vacchi, Andreina Manfredi, Giulia Cassone, Carlo Salvarani, Stefania Cerri, Marco Sebastiani

**Affiliations:** ^1^Rheumatology Unit, Azienda Policlinico di Modena, University of Modena and Reggio Emilia, Modena, Italy; ^2^Ph.D. Program in Clinical and Experimental Medicine, University of Modena and Reggio Emilia, Modena, Italy; ^3^Rheumatology Unit, Azienda USL-IRCCS Reggio Emilia, Reggio Emilia, Italy; ^4^Respiratory Diseases Unit, Azienda Policlinico di Modena, University of Modena and Reggio Emilia, Modena, Italy

## Abstract

Rheumatoid arthritis (RA) is a chronic, systemic, inflammatory disease characterized by joint and extra-articular involvement. Among them, interstitial lung disease (ILD) is one of the most common and severe extra-articular manifestations, with a negative impact on both therapeutic approach and overall prognosis. ILD can occur at any point of the natural history of RA, sometimes before the appearance of joint involvement. Since no controlled studies are available, the therapeutic approach to RA-ILD is still debated and based on empirical approaches dependent on retrospective studies and case series. Here, we report the case of a 75-year-old patient affected by RA complicated by ILD successfully treated with a combination therapy of an antifibrotic agent, nintedanib, and an inhibitor of IL-6 receptor, sarilumab. We obtained a sustained remission of the joint involvement and, simultaneously, a stabilization of the respiratory symptoms and function, with a good safety profile. To date, this is the first report describing a combination therapy with nintedanib and a disease-modifying antirheumatic drug (DMARD) for the management of RA complicated by ILD. Future prospective studies are needed to better define efficacy and safety of this approach in the treatment of these subjects.

## 1. Introduction

Rheumatoid arthritis (RA) is a chronic, systemic, inflammatory disease affecting 0.5–1% of the population worldwide. It is characterized by chronic, symmetrical, erosive synovitis and sometimes by extra-articular manifestations [1]. Among them, lung involvement is common and includes a wide spectrum of disorders ranging from airways and pleural disease, bronchiectasis and nodules, to infection and drug toxicity [1].

Interstitial lung disease (ILD) is the most common lung involvement, with an estimated prevalence ranging from 4 to 30%, and significantly affects the therapeutic approach, quality of life, morbidity, and mortality of RA patients [2, 3]. The treatment of RA-ILD is still debated, and it is mainly based on corticosteroids and immunosuppressants [4]. Usual interstitial pneumonia is the more frequent ILD pattern observed in RA, followed by nonspecific interstitial pneumonia (more typical of connective tissue disease, CTD). Although RA-UIP is frequently reported to have a better prognosis than idiopathic pulmonary fibrosis (IPF), its role on the prognosis of RA-patients is not yet well defined [5].

Nintedanib is a small molecule triple tyrosine-kinase inhibitor approved as an antifibrotic agent for the treatment of IPF [6]. Nintedanib has shown a significant efficacy in reducing the annual rate of decline of forced vital capacity (FVC) in subjects with IPF in comparison with placebo [6] and very recently showed efficacy also in the treatment of fibrosing ILD different by IPF [7].

Here, we present the case of a patient with RA-ILD treated with nintedanib in association with a biologic antirheumatic drug.

## 2. Case Report

A 75-year-old man, former smoker (40 pack-years, until 2007), was referred to the Respiratory Unit of our university hospital because of the appearance of a persistent and productive cough associated with worsening dyspnea on exertion in November 2016. His past clinical history revealed the presence of ischemic heart disease treated with triple percutaneous transluminal coronary angioplasty, type 2 diabetes mellitus, systemic arterial hypertension, and benign prostatic hyperplasia.

He underwent high-resolution computer tomography (HRCT) with the detection of reticular ILD characterized by bibasal thickening of the interstice and interlobular septa associated with traction bronchiectasis.

At the time of diagnosis, FVC was normal (FVC 109%), while the diffusion capacity for carbon monoxide test (DLCO) was severely reduced (DLCO Sb 35%). Echocardiography was not suggestive for pulmonary arterial hypertension. The patient showed digital clubbing at physical examination and chest auscultation revealed velcro crackles.

For the detection of low-titer anti-citrullinated peptides antibodies (ACPA) (89 UI/mm), he was referred to our Rheumatology Unit. The patient did not complain arthritis, sicca syndrome, Raynaud phenomenon, or other symptoms, or sign related to inflammatory arthritis, or CTDs. Both Schirmer test and nailfold capillaroscopy were negative. Erythrocyte sedimentation rate (ESR), C-reactive protein (CRP), antinuclear antibodies (ANA) including extractable nuclear antigen (ENA), and anti-granulocyte antibodies (ANCA), and rheumatoid factor (RF) were all negative.

A surgical lung biopsy confirmed the presence of a UIP pattern, showing an altered architecture due to “honeycombing” aspects accompanied by mild to moderate chronic interstitial inflammation. A diagnosis of IPF was performed, and the patient began a treatment with nintedanib (150 mg twice daily), which was well tolerated.

In December 2017, he was newly referred to our Rheumatology Unit for the appearance of inflammatory arthralgias involving small joints of the hands and swelling of the left wrist associated with an increase in ESR and CRP (30 mm/h and 24 mg/l, respectively) and the presence of rheumatoid factor (174 IU/ml) besides ACPA. The disease activity score in 28 joints (DAS-28-CRP) was 3.96 (moderate disease activity). Ultrasound confirmed the presence of bilateral arthritis of the wrists with power-Doppler positivity. A diagnosis of RA was performed according to 2010 ACR/EULAR criteria [8], and we started a low-dose steroid therapy (methylprednisolone 4 mg/daily) and hydroxycloroquine (200 mg twice daily), in association with nintedanib, was decreased to 100 mg twice daily because of the appearance of drug-related diarrhea.

In February 2019, an arthritis relapse occurred involving small joints of the hands and the knees. ESR and CRP (36 mm/h and 21 mg/l, respectively) increased again (DAS 28 6.71).

**Figure 1:**
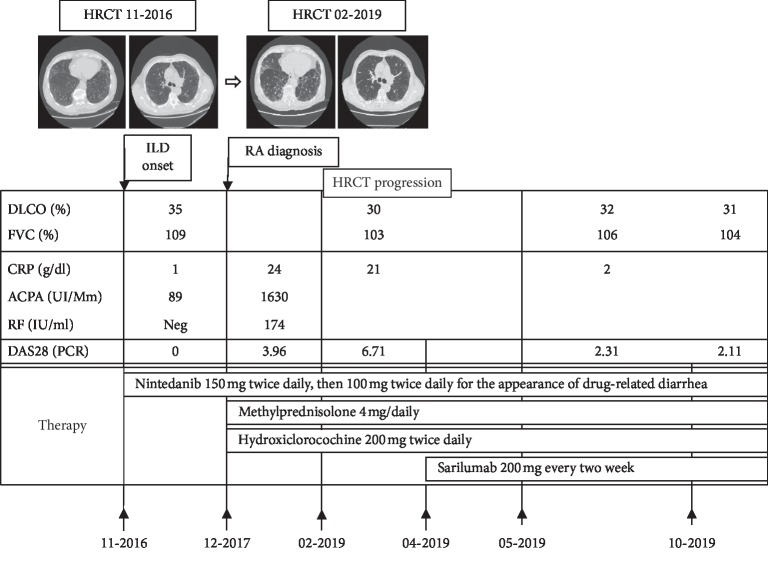
Clinical case summary. HRCT: high-resolution computer tomography; ILD: interstitial lung disease; RA: rheumatoid arthritis; FVC: forced vital capacity; DLCO: diffusing capacity for carbon monoxide test; CRP: c-reactive protein; ACPA: anti-citrullinated peptides antibodies; RF: rheumatoid factor; DAS 28-PCR: disease activity score on 28 joints.

A suspected progression of lung fibrosis at HRCT was also observed, with an increase of fibrosis in the midbasal and in the subpleural and periscissural areas, and an increase in traction bronchiectasis and honeycombing aspects, even if symptoms and respiratory function remained stable (Figure 1). X-rays of the hands showed typical bone erosions in several metacarpophalangeal joints.

In April 2019, indeed, we proposed the association with anti-IL6 receptor antagonist sarilumab (200 mg every two week) achieving a regression of articular involvement (DAS 28-PCR 2.31 one month after), that persisted for 6 months.

## 3. Discussion

ILD has a significant impact on morbidity and mortality and represents a current challenge in the therapeutic approach to RA. Treatment, mainly based on corticosteroids and immunosuppressants [4], is not well defined and efficacy data are not still available. On the other hand, the supposed role of many conventional and biologic disease-modifying antirheumatic drugs (DMARDs) in the onset or worsening of preexisting ILD in RA further complicates the therapeutic choice for these patients [3, 9, 10].

Nintedanib is an antifibrotic agent approved for the treatment of IPF, and recently its efficacy has been demonstrated also in secondary forms of fibrosing ILD [6, 7]. It inhibits the signal transmission at vascular endothelial growth factor receptors, platelet-derived growth factor receptors, and fibroblast growth factor receptors associated with proliferation, migration, and transformation of fibroblasts [6].

There is an increasing interest about the possible role of nintedanib in the treatment of progressive ILD other than IPF. Because of clinical and radiological similarity between RA-ILD and IPF, it is expected that nintedanib could produce similar effects in slowing the progression of the disease. Preclinical data support a possible effect of nintedanib in fundamental processes of lung fibrosis, and that the antifibrotic activity is independent of the cause of the fibrosing lung disease [11–14]. Furthermore, the frequent association between UIP and RA allows us to speculate that nintedanib might also have efficacy in RA-ILD [15, 16].

A randomized, double-blind, placebo-controlled, phase III trial (INBUILD trial) recently assessed the efficacy and safety of nintedanib (150 mg twice daily) versus placebo in 663 patients with a diagnosis of ILD other than IPF, including subjects affected by RA [17]. Patients eligible for the study presented features of diffuse fibrosing lung disease affecting more than 10% of lung volume on HRCT; moreover, they showed disease progression in the last two years before screening, according to FVC, respiratory symptoms, or HRCT. In particular, the study population included a subgroup of patients with UIP-like pattern, namely, patients with reticular abnormalities and traction bronchiectasis with or without honeycombing on HRCT (412 patients, 62.1%) [7].

The patients who received nintedanib showed a slower progression of ILD compared to placebo, as shown by lower decline in the annual rate of FVC over the 52-week period, both in overall population and in UIP-like fibrotic patterns group. Interestingly, the absolute treatment effect of nintedanib in this study was similar in magnitude to those observed in pooled data from the INPULSIS trial [7].

To our knowledge, this is the second report of a patient with RA-ILD treated with nintedanib, and for the first time, we describe the association between nintedanib and a biologic DMARD.

Kakuwa et al. described, for the first time, a 74-year-old man presenting the UIP pattern at HRCT who was diagnosed with IPF and subsequently developed an inflammatory articular involvement typical of RA, with positivity of ACPA and RF. The patient was successfully treated with nintedanib [18].

In our patient, treatment with nintedanib allowed maintenance of lung function along the follow-up period, despite that HRCT showed a progression of fibrotic features of disease. On the other side, we obtained the remission of the joint involvement, observing a good safety profile of the combination therapy, without observing any other side effects. Finally, we need data from specific controlled trials to evaluate the safety and effectiveness of nintedanib in RA-related ILD [7, 18] and the possible association with conventional, biological, and targeted-synthetic DMARDs.
